# (*S*)-2-[(*S*,*E*)-4-(4-Chloro­phen­yl)-1-nitro­but-3-en-2-yl]cyclo­hexa­none

**DOI:** 10.1107/S1600536809029213

**Published:** 2009-07-29

**Authors:** Zhaobo Li, Yi Guo, Bailin Li, Shuping Luo

**Affiliations:** a State Key Laboratory Breeding Base of Green Chemistry-Synthesis Technology, Zhejiang University of Technology, Hangzhou 310014, People’s Republic of China; b Department of Pharmaceutical and Chemical Engineering, Taizhou College, Linhai, Zhejiang 317000, People’s Republic of China

## Abstract

The title compound, C_16_H_18_ClNO_3_, was obtained by the organocatalytic asymmetric Michael addition of cyclo­hexa­none to 1-chloro-4-[(1*E*,3*E*)-4-nitro­buta-1,3-dien­yl]benzene. The double bond has an *E* configuration. The cyclo­hexa­none ring adopts a chair conformation. The conformation of the mol­ecule is stabilized by a weak intra­molecular C—H⋯O hydrogen bond.

## Related literature

For asymmetric Michael addition reactions employing chiral organo­catalysts, see: Belot *et al.* (2008 ▶); Dalko & Moisan (2004 ▶); Yu *et al.* (2009 ▶). For details of the synthesis, see: Xu *et al.* (2008 ▶); For puckering parameters, see: Cremer & Pople (1975 ▶).
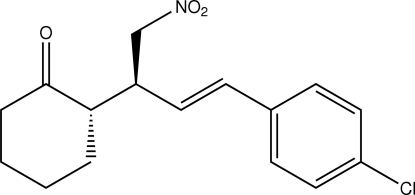


## Experimental

### 

#### Crystal data

C_16_H_18_ClNO_3_
*M* *_r_* = 307.78
Orthorhombic, 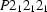

*a* = 5.5300 (3) Å
*b* = 8.5175 (6) Å
*c* = 34.0903 (18) Å
*V* = 1605.71 (17) Å^3^
*Z* = 4
Mo *K*α radiation
μ = 0.25 mm^−1^
*T* = 296 K
0.48 × 0.32 × 0.28 mm

#### Data collection

Rigaku R-AXIS RAPID diffractometer
Absorption correction: multi-scan (*ABSCOR*; Higashi, 1995 ▶) *T*_min_ = 0.879, *T*_max_ = 0.933
15644 measured reflections
2148 independent reflections
1391 reflections with *F* ^2^ > 2σ(*F* ^2^)
*R*_int_ = 0.034

#### Refinement

*R*[*F* ^2^ > 2σ(*F* ^2^)] = 0.044
*wR*(*F* ^2^) = 0.124
*S* = 1.00
2148 reflections
192 parameters
H-atom parameters constrained
Δρ_max_ = 0.22 e Å^−3^
Δρ_min_ = −0.27 e Å^−3^
Absolute structure: Flack (1983 ▶), 1486 Friedel pairs
Flack parameter: 0.27 (18)

### 

Data collection: *PROCESS-AUTO* (Rigaku, 2006 ▶); cell refinement: *PROCESS-AUTO*; data reduction: *CrystalStructure* (Rigaku, 2007 ▶); program(s) used to solve structure: *SIR97* (Altomare *et al.*, 1999 ▶); program(s) used to refine structure: *SHELXL97* (Sheldrick, 2008 ▶); molecular graphics: *ORTEP-3 for Windows* (Farrugia, 1997 ▶); software used to prepare material for publication: *CrystalStructure*.

## Supplementary Material

Crystal structure: contains datablocks global, I. DOI: 10.1107/S1600536809029213/bx2224sup1.cif

Structure factors: contains datablocks I. DOI: 10.1107/S1600536809029213/bx2224Isup2.hkl

Additional supplementary materials:  crystallographic information; 3D view; checkCIF report

## Figures and Tables

**Table 1:** Hydrogen-bond geometry (Å, °)

*D*—H⋯*A*	*D*—H	H⋯*A*	*D*⋯*A*	*D*—H⋯*A*
C8—H81⋯O1	0.97	2.37	3.020 (4)	124

**Fig. 1.**
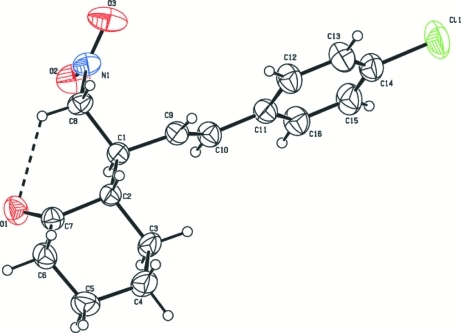
The asymmetric unit of the title compound with the atomic labeling scheme; displacement ellipsoids are drawn at the 30% probability level. Dotted lines show hydrogen bonding.

## supplementary crystallographic information

### Comment

As one of the most important chiral carbon-carbon bond-forming processes in modern organic chemistry, the field of asymmetric Michael addition employing chiral organocatalysts has gained more and more attention and become the focus of intense research efforts (Dalko & Moisan, 2004; Belot *et al.*, 2008; Yu *et al.*, 2009). Consequently, we have synthesized a series of Michael adducts by employing *cyclo*-ketones to nitrodienes in our laboratory. We report here the crystal structure and the absolute configuration of the title compound, (I). The cyclohexanone ring adopts a chair conformation as shown by the Cremer & Pople (1975) puckering parameters [Q_T _= 0.568 (4) Å , θ=3.9 (4)° , φ=355 (6) °]. The C1—C9—C10—C11 torsion angle of 175.2 (3) confirms the *E* configuration of the molecule with respect to the C9═C10 bond. The conformation of (I) is stabilized by one weak intramolecular C—H···O hydrogen bond, Table 1, Fig 1.

### Experimental

A 1,2-dichloroethane (0.5 ml) solution of cyclohexanone (0.25 mmol) and 1-chloro-4-((1*E*,3*E*)-4-nitrobuta-1,3-dienyl)benzene (0.25 mmol) in the presence of (*S*)-1-methyl-2-(pyrrolidin-2-ylmethylthio)-1*H*-imidazole (0.025 mmol) as amine catalyst and (*R*)-2-(3-(3,5-bis(trifluoromethyl)phenyl)thioureido)-2-phenylacetic acid (0.025 mmol) as acid module at room tempreture was stirred vigorously (Xu *et al.*, 2008). After completion of the reaction, the resulted reaction mixture was purified directly by silica gel column chromatography (eluent: petroleum ether-EtOAc). Single crystals were obtained by slow evaporation of an ethanol-dichloromethane solution.

### Refinement

All carbon-bonded H atoms were placed in calculated positions with C—H = 0.93 Å (aromatic), C—H = 0.98 Å (*sp^2^*), C—H = 0.97 Å (*sp*^3^) and refined using a riding model, with *U*_iso_(H)=1.2_eq_(C).

The absolute configuration of this compound is established from both the diffraction data and the absolute configuration of a similar compound reported in Xu *et al.* (2008), therein the organocatalyst has the same structure as in the title compound.

### Crystal data

**Table:**

C_16_H_18_ClNO_3_	*F*(000) = 648.00
*M**_r_* = 307.78	*D*_x_ = 1.273 Mg m^−^^3^
Orthorhombic, *P*2_1_2_1_2_1_	Mo *K*α radiation, λ = 0.71075 Å
Hall symbol: P 2ac 2ab	Cell parameters from 9533 reflections
*a* = 5.5300 (3) Å	θ = 3.0–27.4°
*b* = 8.5175 (6) Å	µ = 0.25 mm^−^^1^
*c* = 34.0903 (18) Å	*T* = 296 K
*V* = 1605.71 (17) Å^3^	Chunk, colourless
*Z* = 4	0.48 × 0.32 × 0.28 mm

### Data collection

**Table:**

Rigaku R-AXIS RAPID diffractometer	1391 reflections with *F*^2^ > 2σ(*F*^2^)
Detector resolution: 10.00 pixels mm^-1^	*R*_int_ = 0.034
ω scans	θ_max_ = 27.4°
Absorption correction: multi-scan (*ABSCOR*; Higashi, 1995)	*h* = −7→6
*T*_min_ = 0.879, *T*_max_ = 0.933	*k* = −11→11
15644 measured reflections	*l* = −43→44
2148 independent reflections	

### Refinement

**Table:**

Refinement on *F*^2^	(Δ/σ)_max_ = 0.001
*R*[*F*^2^ > 2σ(*F*^2^)] = 0.044	Δρ_max_ = 0.22 e Å^−^^3^
*wR*(*F*^2^) = 0.124	Δρ_min_ = −0.27 e Å^−^^3^
*S* = 1.00	Extinction correction: *SHELXL97* (Sheldrick, 2008)
2148 reflections	Extinction coefficient: 0.027 (2)
192 parameters	Absolute structure: Flack (1983), 1486 Friedel pairs
H-atom parameters constrained	Flack parameter: 0.27 (18)
*w* = 1/[σ^2^(*F*_o_^2^) + (0.032*P*)^2^ + 1.2*P*] where *P* = (*F*_o_^2^ + 2*F*_c_^2^)/3	

### Special details

**Table:**

Geometry. ENTER SPECIAL DETAILS OF THE MOLECULAR GEOMETRY
Refinement. Refinement using all reflections. The weighted *R*-factor (*wR*) and goodness of fit (*S*) are based on *F*^2^. *R*-factor (gt) are based on *F*. The threshold expression of *F*^2^ > 2.0 σ(*F*^2^) is used only for calculating *R*-factor (gt).

### Fractional atomic coordinates and isotropic or equivalent isotropic displacement parameters (Å^2^)

**Table:**

	*x*	*y*	*z*	*U*_iso_*/*U*_eq_	
Cl1	0.6163 (3)	0.6280 (2)	1.07164 (3)	0.1211 (6)	
O1	0.9458 (5)	0.1436 (3)	0.77121 (8)	0.0735 (8)	
O2	1.2699 (5)	0.5169 (4)	0.83637 (10)	0.0841 (9)	
O3	0.9882 (6)	0.6855 (3)	0.84395 (12)	0.1019 (12)	
N1	1.0607 (6)	0.5581 (4)	0.83299 (10)	0.0633 (8)	
C1	0.8554 (6)	0.3047 (4)	0.84336 (9)	0.0497 (8)	
C2	0.6749 (6)	0.1873 (4)	0.82559 (9)	0.0486 (7)	
C3	0.6291 (9)	0.0456 (4)	0.85244 (10)	0.0705 (12)	
C4	0.4412 (9)	−0.0647 (5)	0.83580 (12)	0.0801 (13)	
C5	0.5077 (9)	−0.1218 (5)	0.79532 (12)	0.0764 (12)	
C6	0.5632 (7)	0.0143 (4)	0.76796 (10)	0.0643 (10)	
C7	0.7474 (7)	0.1200 (4)	0.78612 (10)	0.0532 (8)	
C8	0.8838 (7)	0.4463 (4)	0.81622 (10)	0.0562 (9)	
C9	0.7707 (7)	0.3624 (4)	0.88279 (9)	0.0552 (8)	
C10	0.8919 (7)	0.3590 (4)	0.91561 (10)	0.0601 (9)	
C11	0.8194 (7)	0.4250 (4)	0.95375 (10)	0.0596 (9)	
C12	0.6144 (9)	0.5173 (5)	0.95838 (12)	0.0754 (12)	
C13	0.5508 (9)	0.5783 (5)	0.99460 (12)	0.0809 (13)	
C14	0.6932 (9)	0.5491 (5)	1.02636 (12)	0.0754 (12)	
C15	0.8973 (10)	0.4591 (6)	1.02286 (12)	0.0837 (14)	
C16	0.9577 (8)	0.3972 (5)	0.98675 (11)	0.0726 (12)	
H1	1.0127	0.2531	0.8464	0.060*	
H2	0.5206	0.2421	0.8221	0.058*	
H9	0.6159	0.4048	0.8839	0.066*	
H10	1.0415	0.3092	0.9148	0.072*	
H12	0.5177	0.5386	0.9367	0.090*	
H13	0.4118	0.6389	0.9972	0.097*	
H15	0.9943	0.4398	1.0446	0.100*	
H16	1.0952	0.3350	0.9846	0.087*	
H31	0.7795	−0.0114	0.8558	0.085*	
H32	0.5737	0.0833	0.8777	0.085*	
H41	0.4259	−0.1546	0.8531	0.096*	
H42	0.2875	−0.0101	0.8344	0.096*	
H51	0.3737	−0.1816	0.7847	0.092*	
H52	0.6493	−0.1886	0.7972	0.092*	
H61	0.6255	−0.0263	0.7434	0.077*	
H62	0.4162	0.0732	0.7631	0.077*	
H81	0.9403	0.4111	0.7908	0.067*	
H82	0.7287	0.4981	0.8133	0.067*	

### Atomic displacement parameters (Å^2^)

**Table:**

	*U*^11^	*U*^22^	*U*^33^	*U*^12^	*U*^13^	*U*^23^
Cl1	0.1546 (14)	0.1453 (13)	0.0635 (6)	−0.0040 (13)	0.0179 (8)	−0.0299 (7)
O1	0.0658 (17)	0.0833 (19)	0.0716 (16)	−0.0074 (17)	0.0162 (14)	−0.0129 (15)
O2	0.0494 (16)	0.086 (2)	0.117 (2)	−0.0064 (16)	−0.0101 (17)	0.006 (2)
O3	0.087 (2)	0.0605 (17)	0.158 (3)	−0.005 (2)	−0.018 (2)	−0.023 (2)
N1	0.0585 (19)	0.0535 (18)	0.078 (2)	−0.0089 (18)	−0.0045 (18)	0.0029 (17)
C1	0.0521 (19)	0.0498 (18)	0.0472 (16)	−0.0037 (18)	−0.0020 (16)	−0.0009 (15)
C2	0.0516 (18)	0.0480 (18)	0.0462 (15)	−0.0035 (17)	0.0013 (15)	−0.0015 (14)
C3	0.101 (3)	0.061 (2)	0.0486 (18)	−0.032 (2)	0.001 (2)	0.0005 (17)
C4	0.109 (3)	0.067 (2)	0.064 (2)	−0.037 (2)	0.000 (2)	−0.003 (2)
C5	0.095 (3)	0.058 (2)	0.076 (2)	−0.010 (2)	−0.009 (2)	−0.009 (2)
C6	0.072 (2)	0.068 (2)	0.0534 (19)	−0.009 (2)	−0.0023 (19)	−0.0126 (18)
C7	0.057 (2)	0.053 (2)	0.0501 (17)	0.0003 (19)	−0.0022 (17)	−0.0014 (16)
C8	0.060 (2)	0.050 (2)	0.0583 (19)	−0.014 (2)	−0.0097 (18)	0.0038 (16)
C9	0.058 (2)	0.055 (2)	0.0526 (17)	−0.0046 (19)	−0.0029 (17)	−0.0023 (17)
C10	0.063 (2)	0.064 (2)	0.0537 (18)	0.005 (2)	−0.0053 (18)	−0.0025 (17)
C11	0.064 (2)	0.060 (2)	0.0547 (19)	−0.005 (2)	−0.0032 (18)	−0.0040 (17)
C12	0.079 (2)	0.089 (3)	0.058 (2)	0.010 (2)	−0.006 (2)	−0.006 (2)
C13	0.085 (3)	0.089 (3)	0.069 (2)	0.014 (3)	0.002 (2)	−0.012 (2)
C14	0.090 (3)	0.079 (2)	0.058 (2)	−0.012 (3)	0.006 (2)	−0.007 (2)
C15	0.097 (3)	0.101 (3)	0.053 (2)	−0.006 (3)	−0.011 (2)	−0.005 (2)
C16	0.075 (2)	0.085 (3)	0.059 (2)	0.004 (2)	−0.011 (2)	−0.003 (2)

### Geometric parameters (Å, °)

**Table:**

Cl1—C14	1.736 (4)	C14—C15	1.370 (7)
O1—C7	1.226 (4)	C15—C16	1.380 (5)
O2—N1	1.214 (4)	C1—H1	0.980
O3—N1	1.216 (4)	C2—H2	0.980
N1—C8	1.480 (5)	C3—H31	0.970
C1—C2	1.537 (4)	C3—H32	0.970
C1—C8	1.528 (4)	C4—H41	0.970
C1—C9	1.506 (4)	C4—H42	0.970
C2—C3	1.536 (5)	C5—H51	0.970
C2—C7	1.516 (4)	C5—H52	0.970
C3—C4	1.512 (6)	C6—H61	0.970
C4—C5	1.509 (5)	C6—H62	0.970
C5—C6	1.519 (5)	C8—H81	0.970
C6—C7	1.494 (5)	C8—H82	0.970
C9—C10	1.305 (4)	C9—H9	0.930
C10—C11	1.472 (5)	C10—H10	0.930
C11—C12	1.389 (6)	C12—H12	0.930
C11—C16	1.381 (5)	C13—H13	0.930
C12—C13	1.385 (6)	C15—H15	0.930
C13—C14	1.362 (6)	C16—H16	0.930
			
O2—N1—O3	122.9 (3)	C2—C3—H32	108.8
O2—N1—C8	118.7 (3)	C4—C3—H31	108.8
O3—N1—C8	118.4 (3)	C4—C3—H32	108.8
C2—C1—C8	110.0 (2)	H31—C3—H32	109.5
C2—C1—C9	111.2 (2)	C3—C4—H41	108.8
C8—C1—C9	108.3 (2)	C3—C4—H42	108.8
C1—C2—C3	112.5 (2)	C5—C4—H41	108.8
C1—C2—C7	115.1 (2)	C5—C4—H42	108.8
C3—C2—C7	106.0 (2)	H41—C4—H42	109.5
C2—C3—C4	112.2 (3)	C4—C5—H51	109.0
C3—C4—C5	112.1 (3)	C4—C5—H52	109.0
C4—C5—C6	111.4 (3)	C6—C5—H51	109.0
C5—C6—C7	110.1 (3)	C6—C5—H52	109.0
O1—C7—C2	122.9 (3)	H51—C5—H52	109.5
O1—C7—C6	122.5 (3)	C5—C6—H61	109.3
C2—C7—C6	114.5 (3)	C5—C6—H62	109.3
N1—C8—C1	110.0 (2)	C7—C6—H61	109.3
C1—C9—C10	126.8 (3)	C7—C6—H62	109.3
C9—C10—C11	127.5 (3)	H61—C6—H62	109.5
C10—C11—C12	122.6 (3)	N1—C8—H81	109.3
C10—C11—C16	120.2 (3)	N1—C8—H82	109.3
C12—C11—C16	117.2 (3)	C1—C8—H81	109.3
C11—C12—C13	121.4 (4)	C1—C8—H82	109.3
C12—C13—C14	119.6 (4)	H81—C8—H82	109.5
Cl1—C14—C13	119.6 (3)	C1—C9—H9	116.6
Cl1—C14—C15	119.7 (3)	C10—C9—H9	116.6
C13—C14—C15	120.6 (4)	C9—C10—H10	116.2
C14—C15—C16	119.4 (4)	C11—C10—H10	116.2
C11—C16—C15	121.8 (4)	C11—C12—H12	119.3
C2—C1—H1	109.1	C13—C12—H12	119.3
C8—C1—H1	109.1	C12—C13—H13	120.2
C9—C1—H1	109.1	C14—C13—H13	120.2
C1—C2—H2	107.6	C14—C15—H15	120.3
C3—C2—H2	107.6	C16—C15—H15	120.3
C7—C2—H2	107.6	C11—C16—H16	119.1
C2—C3—H31	108.8	C15—C16—H16	119.1
			
O2—N1—C8—C1	64.7 (4)	C3—C4—C5—C6	−52.9 (5)
O3—N1—C8—C1	−112.9 (3)	C4—C5—C6—C7	52.5 (5)
C2—C1—C8—N1	−179.0 (2)	C5—C6—C7—O1	118.3 (4)
C8—C1—C2—C3	−177.3 (3)	C5—C6—C7—C2	−58.0 (4)
C8—C1—C2—C7	61.1 (3)	C1—C9—C10—C11	175.2 (3)
C2—C1—C9—C10	126.2 (4)	C9—C10—C11—C12	−7.3 (6)
C9—C1—C2—C3	−57.3 (4)	C9—C10—C11—C16	173.2 (4)
C9—C1—C2—C7	−178.8 (2)	C10—C11—C12—C13	−179.8 (4)
C8—C1—C9—C10	−112.8 (4)	C10—C11—C16—C15	179.0 (4)
C9—C1—C8—N1	59.2 (3)	C12—C11—C16—C15	−0.6 (6)
C1—C2—C3—C4	176.8 (3)	C16—C11—C12—C13	−0.2 (6)
C1—C2—C7—O1	7.5 (5)	C11—C12—C13—C14	0.8 (7)
C1—C2—C7—C6	−176.2 (3)	C12—C13—C14—Cl1	179.0 (3)
C3—C2—C7—O1	−117.6 (4)	C12—C13—C14—C15	−0.5 (7)
C3—C2—C7—C6	58.7 (4)	Cl1—C14—C15—C16	−179.8 (3)
C7—C2—C3—C4	−56.6 (4)	C13—C14—C15—C16	−0.3 (6)
C2—C3—C4—C5	56.4 (4)	C14—C15—C16—C11	0.8 (7)

### Hydrogen-bond geometry (Å, °)

**Table:**

*D*—H···*A*	*D*—H	H···*A*	*D*···*A*	*D*—H···*A*
C8—H81···O1	0.97	2.37	3.020 (4)	124
